# Pentagalloylglucose Inhibits Melanogenesis via Suppression of MITF Signaling Pathway

**DOI:** 10.3390/ijms26104861

**Published:** 2025-05-19

**Authors:** Jung-Wook Kang, In-Chul Lee

**Affiliations:** 1 College of Fusion and Convergence, Seowon University, Cheongju 28674, Republic of Korea; jwkkang@gmail.com; 2 Department of Cosmetic Science and Technology, Seowon University, Cheongju 28674, Republic of Korea

**Keywords:** pentagalloylglucose, melanogenesis, MITF, tyrosinase, α-MSH

## Abstract

Pentagalloylglucose (PGG) is a powerful antioxidant and a naturally derived polyphenolic compound present in tannins. In this study, we investigated the ability of PGG to selectively inhibit hyperpigmentation through the regulation of melanogenesis in melanocytes. PGG inhibited melanin production in α-melanocyte-stimulating hormone (α-MSH)-induced B16F10 melanoma cells. Furthermore, PGG suppressed the expression of melanin synthesis enzymes, such as tyrosinase, tyrosinase-related protein (TRP)-1, and TRP-2. The mRNA and protein expression of the microphthalmia-associated transcription factor, which is involved in the mechanism of melanogenesis, was also reduced by PGG, and this effect was induced via PKA/CREB and MAPK phosphorylation. These results suggest that PGG inhibits α-MSH-induced melanin production by regulating the PKA/CREB/MAPK signaling pathway, indicating that natural compounds can serve as inhibitors of melanogenesis.

## 1. Introduction

Melanin, a crucial pigment involved in skin pigmentation, is produced via melanogenesis [1], a biosynthetic pathway consisting of five major signaling pathways [2]. The regulation of melanogenesis is of great significance for understanding both the physiological role of melanin and its pathological hyperactivity, which contributes to hyperpigmentation disorders. Melanogenesis is controlled by external stimuli, such as UV rays, α-melanocyte-stimulating hormone (α-MSH), stem cell factor (SCF), and nitric oxide, and endogenous factors such as inflammation [2,3]. The melanogenic enzymes tyrosinase, tyrosinase-related protein (TRP)-1, and TRP-2 regulate melanogenesis, and they are transcriptionally modulated by a signaling pathway involving microphthalmia-associated transcription factor (MITF) and several kinases [2,3]. The levels of mediators secreted in response to UV irradiation, such as α-MSH, increase through interactions with their respective cell membrane receptors in melanocytes [4]. Melanocortin 1 receptor (MC1R), the receptor for α-MSH, increases cyclic adenosine monophosphate (cAMP) content by interacting with α-MSH [5]. Increases in intracellular cAMP content leads to the activation of protein kinase A (PKA), which subsequently phosphorylates cAMP response element-binding protein (CREB), an activator of MITF expression, at Ser133 [2,3,4,5]. Activated CREB increases MITF gene expression after binding to a cAMP response element motif located between 140 and 147 bp from the transcriptional site of the MITF gene [6]. SCF binds to c-kit on the cell membrane and regulates the expression of enzymes involved in melanogenesis by activating the mitogen-activated protein kinases (MAPKs) p38, extracellular signal-regulated kinase (ERK), and c-Jun N-terminal kinase (JNK) [7]. Phosphorylated p38, which can also be activated by UV rays, induces MITF expression, stimulating melanogenic enzyme production [8]. Given the central role of MITF in melanogenesis, it serves as a critical target for regulating melanin synthesis. MITF binds to the promoter elements of tyrosinase, TRP-1, and TRP-2 to activate melanin synthesis genes, highlighting its importance in therapeutic strategies against hyperpigmentation disorders [8]. Moreover, targeting key pathways, such as the MC1R/cAMP/PKA/CREB axis or SCF/c-kit/MAPK cascade, could offer effective strategies to modulate melanogenesis. These approaches both provide insights into the physiological regulation of pigmentation and open avenues for addressing pathological conditions such as melasma and freckles. Studies on the efficacy of naturally derived pigmentation inhibition agents are currently ongoing. Tretinoin (all-trans retinoic acid) interferes with melanogenesis and melanocyte activity by transcriptionally regulating tyrosinase [9] *Glycyrrhiza glabra* extracts, containing active components such as glycyrrhizin, triterpene saponins, flavonoids, and glabridin, exert skin-whitening, anti-aging, anti-inflammatory, anti-acne, and photoprotective effects, primarily through antioxidant activity, the inhibition of tyrosinase, and UVB-induced melanogenesis [10]. Kojic acid (5-hydroxy-2-hydroxymethyl-4H-pyran-4-one), which is produced by species such as *Aspergillus* and *Penicillium*, suppresses melanin biosynthesis by binding to copper [11]. Identified as one of the few effective hypopigmenting agents, kojic acid exerts its skin-whitening effects through both the inhibition of tyrosinase activity and suppression of melanocyte proliferation, as demonstrated in clinical studies, highlighting the essential role of melanocyte–keratinocyte crosstalk in the pharmacological mechanisms of dermatological agents [12,13]. This dual focus on understanding the mechanisms and developing potential interventions underscores the translational relevance of melanogenesis research.

Pentagalloylglucose (1,2,3,4,6-penta-O-galloyl-b-d-glucose, PGG) is a representative gallotannin compound named [(2R, 3R, 4S, 5R, 6S)-3,4,5,6-tetrakis [(3,4,5-trihydroxybezoyl) oxy] oxan-2-yl] methyl 3,4,5-trihydroxybenzoate under the International Union of Pure and Applied Chemistry nomenclature [14]. Gallotannins are tannins obtained through the liberation of gallic acid from hydrolyzable tannins, and PGG is a polyphenolic compound in which gallic acid is ester-bonded to all five hydroxyl groups of glucose [15,16]. The plant-derived phenolic compound PGG occurs primarily in the beta form, whereas the anomeric alpha form is rarely found in nature [17]. Because PGG compounds are a type of tannin and are known for their powerful antioxidant activities, they are being studied as potential therapeutic agents [15]. PGG has a stronger ROS-scavenging effect than gallic acid, a hydrolysis product of tannic acid commonly reported in the pharmaceutical, cosmetic, and food industries, because of its higher number of galloyl groups [18].

PGG has potential as a pharmacological agent with therapeutic or preventive effects against various diseases. In addition to its antioxidant properties [17], PGG has been used as a pharmacological agent for several cancers, including hepatocellular carcinoma [19], prostate cancer [20], breast cancer [21], and glioma [22], because of its ability to modulate molecular targets and signaling pathways. PGG is also a beneficial natural phenolic compound with synergistic effects as an adjuvant to treatments such as chemotherapy and radiotherapy [23,24,25]. Although PGG is abundant in nature, natural sources contain slightly different amounts [26]. Marian plum (*Bouea macrophylla*) seeds [27], mango (*Mangifera indica*) seeds [28], and peony (*Paeonia lactiflora*) roots [29] have been found to contain PGGs via solvent-based extraction. PGG is also reported to be a physiologically active compound with numerous potential health effects [14,26]. However, the effects of PGG as a depigmentation agent have not yet been reported. This study investigated the potential of PGG to inhibit hyperpigmentation through melanogenesis regulation. PGG inhibited the PKA/CREB and MAPK signaling pathways in B16F10 cells, inducing anti-melanogenic effects.

## 2. Results

### 2.1. Effects of PGG on Cell Viability and Melanin Content in B16F10 Cells

**Figure 1:**
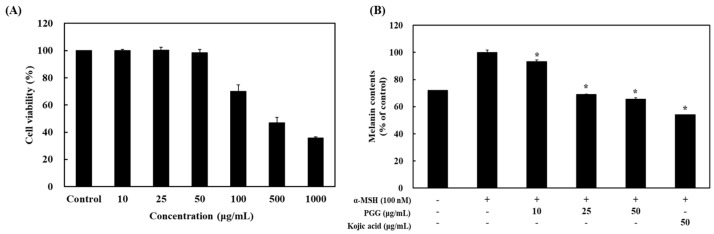
Effects of PGG on cell viability and melanin production. (**A**) B16F10 cells were seeded in 96-well plates (1 × 10^4^ cells/well) and incubated for 24 h. The cells were treated with different concentrations of PGG. Cell viability was measured using the MTT assay. (**B**) The cells were treated with α-MSH (100 nM) and PGG (10, 25, and 50 μg/mL) for 24 h. The treated cells were observed using an ELISA reader at 490 nm. The data are presented as the mean ± SD of three independent experiments. * *p* < 0.05 compared with the α-MSH group. MTT, 3-(4,5-dimethylthiazol-2-yl)-2,5-diphenyltetrazolium bromide. α-MSH: α-melanocyte-stimulating hormone, PGG: pentagalloylglucose, ELISA: enzyme-linked immunosorbent assay; kojic acid: positive control.

α-MSH can stimulate melanogenesis in cultured melanocytes, primarily increasing eumelanin synthesis rather than overall melanin production [30]. In particular, eumelanin contributes to skin darkening, which represents the observed effects of α-MSH [30]. Prior to evaluating the inhibitory effect of PGG following α-MSH exposure, the cytotoxicity of PGG was examined. B16F10 cells were treated with different PGG concentrations for 24 h. PGG did not exert cytotoxic effects at concentrations lower than 50 μg/mL, whereas high concentrations (100, 500, and 1000 μg/mL) induced cytotoxicity in B16F10 melanoma cells (Figure 1). Thus, noncytotoxic concentrations (10, 25, and 50 μg/mL) were used to assess the efficacy of PGG in B16F10 cells. To measure the effect of PGG on melanin production, the cells were treated with 100 nM α-MSH for 24 h and then exposed to various concentrations of PGG. Treatment with PGG significantly suppressed the production of melanin in a concentration-dependent manner. At a concentration of 50 μg/mL, PGG decreased melanin content to 34.46% of that in the α-MSH group. Additionally, kojic acid, a positive control, inhibited melanin production with a similar potency as PGG (Figure 1).

### 2.2. Effects of PGG on Melanogenic Enzyme Expression in B16F10 Cells

**Figure 2:**
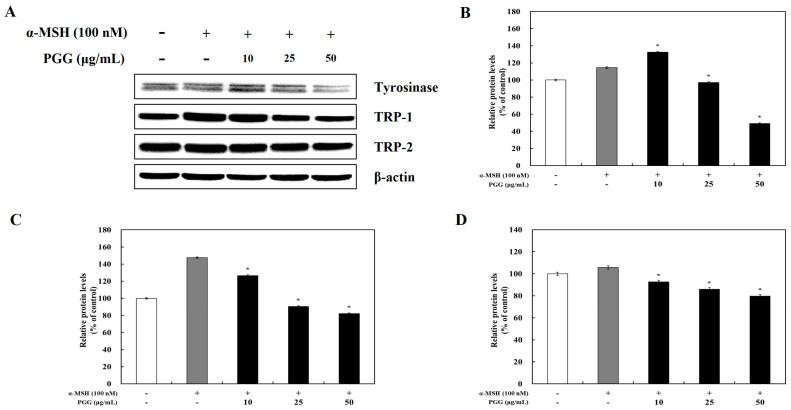
Effects of PGG on the protein expression of melanogenesis-related enzymes in B16F10 cells. Representative western blots (**A**) and relative densitometric quantification of (**B**) tyrosinase, (**C**) TRP-1, and (**D**) TRP-2. Protein expression was measured by western blotting 24 h after α-MSH treatment. The data are presented as the mean ± SD of three independent experiments. * *p* < 0.05 compared with the α-MSH group. GAPDH, glyceraldehyde 3-phosphate dehydrogenase; α-MSH: α-melanocyte-stimulating hormone; PGG: pentagalloylglucose; TRP-1: tyrosinase-related protein-1; TRP-2: tyrosinase-related protein-2.

**Figure 3:**
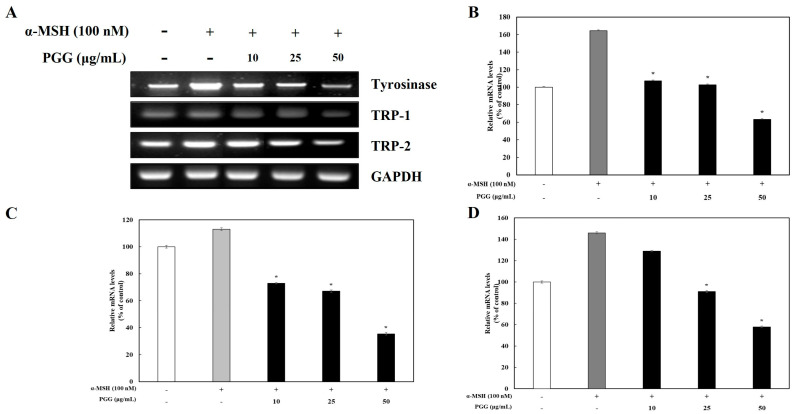
Effects of PGG on the mRNA expression of melanogenesis-related enzymes in B16F10 cells. Representative mRNA expression (**A**) and relative densitometric quantification of (**B**) tyrosinase, (**C**) TRP-1, and (**D**) TRP-2. mRNA expression was detected by RT-PCR 24 h after α-MSH treatment. The data are presented as the mean ± SD of three independent experiments. * *p* < 0.05 compared with the α-MSH group. GAPDH, glyceraldehyde 3-phosphate dehydrogenase; α-MSH: α-melanocyte-stimulating hormone; PGG: pentagalloylglucose; TRP-1: tyrosinase-related protein-1; TRP-2: tyrosinase-related protein-2.

Tyrosinase affects the production of melanin and the formation of other pigments from tyrosine via oxidation [31]. TRP-1 and TRP-2, which are melanogenic enzymes, are gene products specific to melanocytes that participate in melanin synthesis through several enzymatic processes [32]. It is known that tyrosinase and its related melanogenic enzymes participate in melanogenesis. To examine whether tyrosinase, TRP-1, and TRP-2 expression was influenced by PGG production, western blotting and reverse transcription polymerase chain reaction (RT-PCR) were performed. The protein expression of tyrosinase, TRP-1, and TRP-2 was significantly decreased by PGG compared with that in α-MSH-treated cells (Figure 2). Treatment with 50 μg/mL PGG after α-MSH stimulation reduced tyrosinase, TRP-1, and TRP-2 protein expression by 65.34%, 65.40%, and 25.98%, respectively. Additionally, RT-PCR demonstrated that PGG reduced the mRNA expression of tyrosinase, TRP-1, and TRP-2 (Figure 3). Specifically, treatment with 50 μg/mL PGG after α-MSH stimulation reduced tyrosinase, TRP-1, and TRP-2 mRNA expression by 97.19%, 70.61%, and 88.25%, respectively. These results demonstrated that PGG significantly inhibited the expression of melanogenic enzymes.

### 2.3. Effects of PGG on the Protein and mRNA Expression of MITF in B16F10 Cells

**Figure 4:**
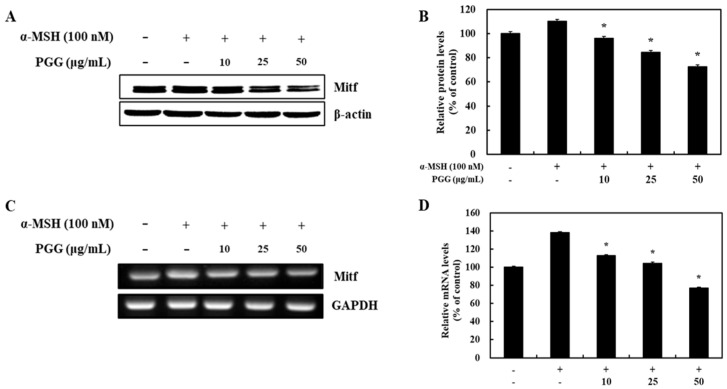
Effects of PGG on MITF expression in B16F10 cells. Representative western blots (**A**) and relative densitometric quantification of (**B**) MITF. Protein expression was measured by western blotting 24 h after α-MSH treatment. mRNA expression (**C**) and relative densitometric quantification of MITF (**D**). mRNA expression was detected by RT-PCR 24 h after α-MSH treatment. The data are presented as the mean ± SD of three independent experiments. * *p* < 0.05 compared with the α-MSH group. GAPDH, glyceraldehyde 3-phosphate dehydrogenase; α-MSH: α-melanocyte-stimulating hormone; PGG: pentagalloylglucose; MITF: microphthalmia-associated transcription factor.

Melanogenesis-related genes including tyrosinase, TRP-1, and TRP-2 are modulated by MITF through transcriptional regulation [33,34]. Because PGG was demonstrated to inhibit melanogenic enzyme production following α-MSH stimulation, the effect of PGG on the protein and mRNA expression of MITF, which is known as the main regulator of pigment biosynthesis, was verified. MITF was activated as part of the melanogenesis signaling pathway following α-MSH stimulation. However, subsequent treatment with PGG decreased both the protein and mRNA expression of MITF in B16F10 cells. Treatment with 50 μg/mL PGG exerted the strongest effect on MITF expression, reducing its protein and mRNA expression by 37.55% and 61.29%, respectively (Figure 4). Thus, these data revealed that the reduction in tyrosinase-related gene expression induced by PGG is related to its effect on MITF expression.

### 2.4. Effect of PGG on the Phosphorylation of PKA/CREB in B16F10 Cells

**Figure 5:**
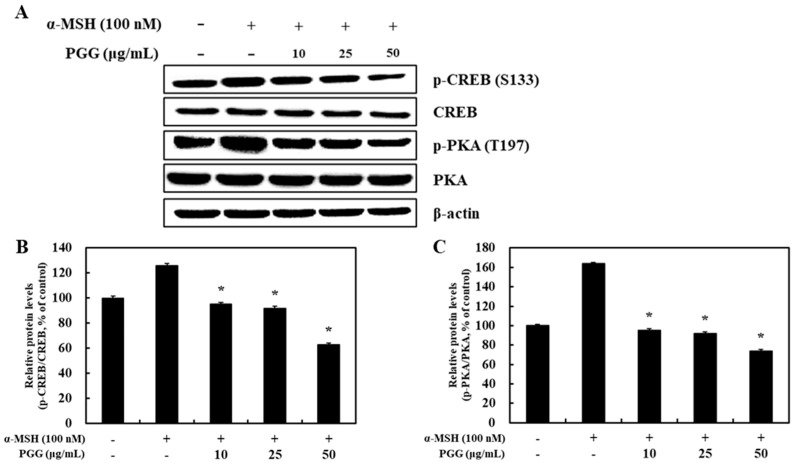
Effects of PGG on the PKA/CREB pathway in B16F10 melanoma cells. Representative western blots (**A**) and relative densitometric quantification of (**B**,**C**) CREB and PKA protein expression, which was measured by western blotting 12 h after α-MSH treatment. The data are presented as the mean ± SD of three independent experiments. * *p* < 0.05 compared with the α-MSH group. α-MSH: α-melanocyte-stimulating hormone; PGG: pentagalloylglucose; CREB: cAMP response element-binding protein; PKA: protein kinase A.

Previous research illustrated that MITF expression is controlled by multiple melanogenesis pathways [35]. In one of these pathways, several kinases that target CREB modulate the transcription of MITF [32,33,34]. It is known that melanogenesis is stimulated by α-MSH, which leads to CREB phosphorylation [36]. Notably, the stimulation of melanogenesis and increases in intracellular cAMP levels reflect the enhancement of PKA activity by phosphorylation [37]. To confirm the regulation of melanogenesis, the effect of PGG on the phosphorylation of PKA/CREB was examined in α-MSH-stimulated B16F10 cells. The phosphorylation of PKA (Thr197) and CREB (Ser133) was triggered by α-MSH treatment. However, the PGG treatment reduced PKA and CREB phosphorylation compared with the effect of α-MSH (Figure 5). The expression level following treatment with PGG (50 μg/mL) decreased to approximately half of the α-MSH-treated group. In addition, CREB phosphorylation was decreased by 63.01% in the same group. In accordance with the protein expression results, PGG appears to be involved in the phosphorylation of proteins in the PKA/CREB signaling pathway.

### 2.5. Effects of PGG on the MAPK Signaling Pathway in B16F10 Cells

**Figure 6:**
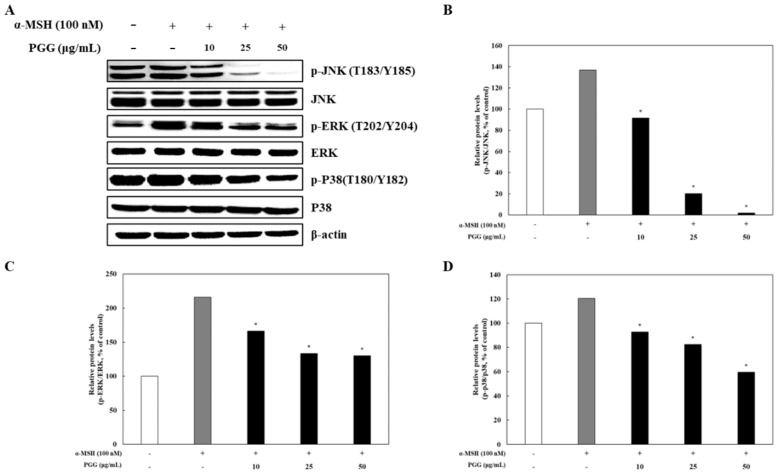
Effects of PGG on the MAPK signaling pathway in B16F10 melanoma cells. Representative western blots (**A**) and relative densitometric quantification of (**B**) p-JNK/JNK, (**C**) p-ERK/ERK, and (**D**) p-p38/p38 protein expression, which was measured by western blotting 12 h after α-MSH treatment. The data are presented as the mean ± SD of three independent experiments. * *p* < 0.05 compared with the α-MSH group. α-MSH: α-melanocyte-stimulating hormone; PGG: pentagalloylglucose; JNK: c-Jun N terminal kinase; ERK: extracellular signal-regulated kinase.

In melanogenesis signaling pathways, α-MSH regulates the production of melanin through modulation of the cAMP/PKA/CREB/MAPK pathway in melanocytes [38]. It has been reported that the MAPK signaling pathway (p38, ERK, and JNK) is mediated by CREB phosphorylation [39]. In addition, the phosphorylation of p38, ERK, and JNK reflects the transcriptional activation of CREB by cAMP and its subsequent binding to MITF promoters [35]. The changes in p38, ERK, and JNK phosphorylation in response to treatment were investigated by western blotting. PGG significantly decreased the phosphorylation of p38, ERK, and JNK in α-MSH-treated B16F10 cells (Figure 6). In particular, the group treated with 50 μg/mL PGG exhibited the greatest reduction in phosphorylated protein levels compared with that induced by α-MSH treatment. These results indicate that PGG suppressed the MAPK signaling pathway in α-MSH-treated B16F10 cells. In summary, PGG induced anti-melanogenic effects by downregulating the PKA/CREB signaling pathways by inhibiting the MAPK signaling pathway in B16F10 melanoma cells.

## 3. Discussion

The skin, being the body’s outermost layer, is one of the most visible and defining features of an individual [40]. It acts as the main defense against external factors, with melanocytes playing a key role in essential functions such as protection against UV radiation and regulation of body temperature by producing melanin. The amount of melanin produced determines the skin color and degree of tanning ability, which are important indicators of an individual’s risk for skin pigmentation within the general population [40,41]. The overproduction of melanin triggered by inflammation or dermatological conditions presents as the accumulation of hyperpigmented spots, in which melanosomes accumulate, leading to darker than normal areas in skin. Eventually, post-inflammatory hyperpigmentation occurs in several skin conditions such as eczema, acne, and contact dermatitis, leading to a negative impact on people’s quality of life [41,42]. Regarding depigmentation agent development, strategies modulating melanogenesis have been considered more promising for greater efficacy and safety. Plant-derived natural compounds have received attention as ideal alternatives for safe and effective therapies. Natural product research for regulating melanogenesis was optimistically considered a new means of safely modulating melanocytes.

Natural products are organic compounds that are biosynthesized and accumulated within organisms such as plants and microorganisms [43]. The secondary metabolites of natural products, including alkaloids, flavonoids, and phenolic compounds, are distinctly distributed in specific organisms, serving as unique components and exhibiting distinctive biological activities [44]. Therefore, these compounds are key components in improving the quality of human life. The confirmed antioxidant properties of phenolic compounds highlight their potential roles in preventing various diseases while demonstrating a broad spectrum of pharmacological and physiological properties [45]. PGG is a plant-derived polyphenol compound and a hydrolyzable tannin that exerts various biological effects through hydrogen bonding with collagen [14]. This study investigated the potential of PGG to inhibit hyperpigmentation via melanogenesis control. According to the results, PGG displayed anti-melanogenesis effects in α-MSH-stimulated B16F10 cells. We focused on evaluating the cytotoxic effects of PGG in the murine melanoma cell line B16F10. While higher concentrations of PGG (≥100 µg/mL) exhibited cytotoxicity, the concentrations used to assess efficacy (10–50 µg/mL) did not compromise cell viability. Furthermore, PGG was confirmed to be safe in human normal dermal fibroblasts, while SK-MEL-2 human melanoma cells exhibited cytotoxic responses comparable to those observed in B16F10 cells (Figure S1). By reducing melanin content without induing cytotoxicity, PGG prevented the accumulation of melanin pigments in a concentration-dependent manner (Figure 2). These results indicated that PGG reduced melanin content at the utilized concentrations without cytotoxicity. The B16F10 cell line is a widely established model for studying melanogenesis due to its relative resistance to certain chemotherapeutic agents. Furthermore, B16F10 cells are known to generate metastatic variants at a high frequency, suggesting that gene amplification mechanisms may contribute to their aggressive metastatic phenotype [46]. Nevertheless, we have confirmed that another compound, feruloylserotonin, inhibits hydrogen peroxide-induced melanogenesis and apoptosis in both B16F10 and SK-Mel-2 melanoma cells [47]. To support the quality of our results, we plan to conduct additional experiments using human melanoma models SK-MEL-2 in future studies.

In melanogenesis, depigmenting mechanisms are involved in the control of various melanin synthesis processes [32]. The mechanisms of melanogenesis modulation by PGG were examined by western blotting and RT-PCR. The expression of melanogenic enzymes following α-MSH stimulation in B16F10 cells led to melanogenesis, whereas PGG treatment reduced their expression (Figure 2 and Figure 3). Specifically, in α-MSH-stimulated B16F10 cells, the enhancement of MITF expression was decreased by PGG, indicating that the increase in melanogenic enzyme expression by α-MSH stimulation is related to MITF expression in melanocytes (Figure 4). In addition, PGG treatment blocked the phosphorylation of PKA at Thr197 and CREB at Ser133 (Figure 5). PGG also significantly decreased the phosphorylation of p38, ERK, and JNK in melanocytes (Figure 6). The PKA/CREB/MAPK signaling pathway in melanocytes leads to melanogenesis by activating MITF [37]. According to reports, α-MSH stimulation leads to the phosphorylation of PKA and increases cAMP levels, which in turn affects CREB signaling [35,36,37]. Furthermore, CREB phosphorylation could be attributable to MAPK signaling pathways, which affects MITF levels in melanocytes [37]. Resveratrol, known as a powerful antioxidant, inhibits melanogenesis by downregulating MITF and tyrosinase expression through ERK signaling, while also decreasing post-transcriptional processing of tyrosinase in melanocyte [48,49]. Similarly, ellagic acid suppresses α-MSH-induced melanogenesis by downregulating the CREB/MITF signaling pathway and its associated proteins in B16F10 cells [50]. In comparison, previous studies have shown that PGG upregulates heme oxygenase-1 (HO-1) expression by promoting Nrf2 nuclear translocation in an ERK-dependent manner in HepG2 cells [51]. Consistent with these findings, we observed that PGG inhibits MITF expression by suppressing both the PKA/CREB and MAPK signaling pathways, suggesting that its potent antioxidant capacity may indirectly contribute to its anti-melanogenic effects.

PGG, a tannin-based non-flavonoid polyphenolic compound, demonstrated potent antioxidant properties, as evidenced by its free radical scavenging effects in DPPH (IC_50_: 11.25 µg/mL) and ABTS (IC_50_: 19.92 µg/mL) assays (Figures S2 and S3). These findings suggest its potential as a multifunctional cosmetic ingredient. In future studies, we plan to investigate whether PGG modulates antioxidant systems in melanoma cells to gain deeper insights into the mechanistic interplay between oxidative stress and melanogenesis.

## 4. Materials and Methods

### 4.1. Chemical

PGG (purity ≥ 98%) was purchased from ChemFaces (Wuhan, China). α-MSH, 3-(4,5-dimethylthiazol-2-yl)-2,5-diphenyltetrazolium bromide (MTT), 2-mercaptoethanol, dimethyl sulfoxide (DMSO), kojic acid, mushroom tyrosinase, l-DOPA, and radioimmunoprecipitation assay buffer (RIPA) were obtained from Sigma-Aldrich (St. Louis, MO, USA). Fetal bovine serum (FBS), Dulbecco’s modified Eagle’s medium (DMEM), 100 U/mL penicillin/streptomycin, and a bicinchoninic acid (BCA) assay kit were purchased from Thermo Fisher Scientific (Waltham, MA, USA). HPLC-grade trifluoroacetic acid, acetonitrile, and methanol were purchased from Fisher Scientific Korea (Seoul, Republic of Korea). Primary antibodies against tyrosinase, TRP-1, TRP-2, MITF, PKA, p-PKA (Thr197), CREB, p-CREB (Ser133), p-ERK1/2 (Thr202/Tyr204), p38, p-p38 (Thr180/Tyr182), JNK, p-JNK (Thr183/Tyr185), and β-actin were purchased from Cell Signaling Technology (Danvers, MA, USA). Horseradish peroxidase (HRP)-conjugated secondary anti-rabbit antibody was obtained from Cell Signaling Technology. ERK 1/2 and anti-mouse (m-IgGκ BP-HRP) were obtained from Santa Cruz Biotechnology, Inc. (Dallas, TX, USA). Immobilon western chemiluminescent HRP substrate for enhanced chemiluminescence was purchased from Merck Millipore (Burlington, MA, USA).

### 4.2. Cell Culture

Murine melanoma cells (B16F10) and human normal dermal fibroblast (CCD-986sk) were procured from American Type Culture Collection (Manassas, VA, USA). SK-MEL-2 cells were purchased from the Korean Cell Line Bank (Seoul, Republic of Korea). B16F10 and CCD-986sk cells were cultured in DMEM supplemented with 10% FBS, 1% penicillin (100 IU/mL), and streptomycin (100 μg/mL). SK-MEL-2 cells were cultured in RPMI 1640 supplemented with 10% FBS, 1% penicillin (100 IU/mL), and streptomycin (100 μg/mL). The cells were maintained at 37 °C in a 5% CO_2_ humidified environment.

### 4.3. Cell Viability

The cells were inoculated into a 96-well microplate at a volume of 100 μL to assess cell viability. After incubation for 24 h, the cells were treated with different concentrations of PGG (up to 1000 μg/mL) for 24 h. Cells were incubated with MTT (2.5 mg/mL) for 4 h. The medium was removed and dissolved in DMSO (100 μg/mL). Cytotoxicity was detected by measuring the absorbance at 540 nm using a microplate reader.

### 4.4. Measurement of Antioxidant Ability

The radical scavenging activity of PGG was evaluated using DPPH and ABTS assays. For the DPPH assay, 180 μM DPPH solution was mixed with serially diluted PGG at a ratio of 1:6 and incubated for 15 min at room temperature. The absorbance was then measured at 517 nm using a microplate reader (Figure S2). For the ABTS assay, 7 mM ABTS solution was reacted with 2.45 mM potassium persulfate for 18 h to generate ABTS cationic radicals. After completion of the reaction, the ABTS solution was diluted with ethanol, and then mixed with PGG solutions (diluted to various concentrations) in a 1:1 ratio. The absorbance was measured at 700 nm (Figure S3).

### 4.5. Measurement of Melanin Content

Melanin content was measured according to a previously described method [52]. B16F10 cells (1 × 10^6^ cells/well) cells were seeded in a 100-mm plate and incubated for 24 h. Afterward, the cells were stimulated with α-MSH (100 nM) in the presence or absence of various concentrations of PGG (10, 25, and 50 μg/mL) and kojic acid (50 μg/mL, positive control) for 24 h. The cells were washed twice with PBS and lysed with lysis buffer (67 mM sodium phosphate buffer, 1% Triton X-100, 0.1 mM phenylmethylsulfonyl fluoride). After centrifuging the cell supernatant, the pellets were dissolved in 1 N NaOH containing 10% DMSO for 1 h at 90 °C. The total protein content was quantified using a BCA assay kit. The absorbance was measured at 405 nm using a microplate reader.

### 4.6. RT-PCR

B16F10 cells were treated with α-MSH (100 nM) in the presence or absence of different concentrations of PGG (10, 25, and 50 µg/mL) for 24 h. The total cellular RNA was isolated using TRIzol (Thermo Fisher Scientific) following the manufacturer’s instructions. RNA was reverse-transcribed using 1 mM dNTPs, oligo dT primers, 5× Green GoTaq Flexi Buffer, and Taq DNA polymerase (Promega, Madison, WI, USA). GAPDH was used to normalize gene expression. The primer sequences of the genes were as follows: MITF, TAGCTCCTTAATGCGGTCGT (reverse) and AGCGTGTATTTTCCCCACAG (forward); tyrosinase, GCCATGACCAGGATGAC (reverse) and GACGGTCACTGCAGACTTTG (forward); TRP-1, AGCTTCCCATCAGATGTCGT (reverse) and ACTTCACTCAAGCCAACTGC (forward); TRP-2, AATGCAGTGGCTTGGAAATC (reverse) and GGCCAAGTGGCTGTAGACC (forward); and GAPDH, AGCCTTCTCCATGGTGGTGAAGAC (reverse) and CGGAGTCAACGGATTTGGTCGTAT (forward).

### 4.7. Western Blotting

B16F10 cells were seeded in a 100-mm plate and cultured for 24 h. The cells were pretreated with PGG (10, 25, and 50 µg/mL) following induction with α-MSH (100 nM). After washing twice, the cells were lysed with cold RIPA [50 mM Tris-HCl, pH 8.0, 150 mM sodium chloride, 1% Igepal CA-630 (NP-40), 0.5% sodium deoxycholate, and 0.1% sodium dodecyl sulfate with protease and phosphatase inhibitor cocktail], buffered for 20 min and centrifuged at 13,200× *g* for 30 min at 4 °C. The total protein content was measured using a BCA kit for standardization. Equal amounts of protein were subjected to 10% sodium dodecyl sulfate-polyacrylamide gel electrophoresis and transferred to polyvinylidene difluoride membranes (Millipore, Billerica, MA, USA). The membranes were blocked with 10% skimmed milk for 1 h at room temperature and incubated overnight with primary antibodies against tyrosinase, TRP-1, TRP-2, MITF, PKA, p-PKA (Thr197), CREB, p-CREB (Ser133), ERK 1/2, p-ERK1/2 (Thr202/Tyr204), p38, p-p38 (Thr180/Tyr182), JNK, p-JNK (Thr183/Tyr185), and β-actin. After washing with Tris-buffered saline with 0.1% Tween 20, the membrane was incubated with HRP-conjugated secondary antibodies for 1 h. The protein bands were detected using enhanced chemiluminescence reagents and quantified using the ChemiDoc imaging system.

### 4.8. Statistical Analysis

All experiments were independently repeated at least three times, and the same experiment was repeated at least three times. The result values were expressed as the mean ± standard deviation of each item. IBM SPSS Statistics (ver. 23, IBM Corp., Armonk, NY, USA) were used to conduct a paired-samples *t*-test (* *p* < 0.05) for significance validation.

## Data Availability

The original contributions presented in this study are included in the article. Further inquiries can be directed to the corresponding author(s).

## Supplementary Materials

The following supporting information can be downloaded at: https://www.mdpi.com/article/10.3390/ijms26104861/s1.
