# Supplementary material for: Pentagalloylglucose Inhibits Melanogenesis via Suppression of MITF Signaling Pathway
Source: Int J Mol Sci. 2025 May 19;26(10):4861. doi: 10.3390/ijms26104861 (PMC12112744; doi:10.3390/ijms26104861)
Supplement: Supplementary file 1 [file ijms-26-04861-s001.zip › ijms-3593851-supplementary.pdf]

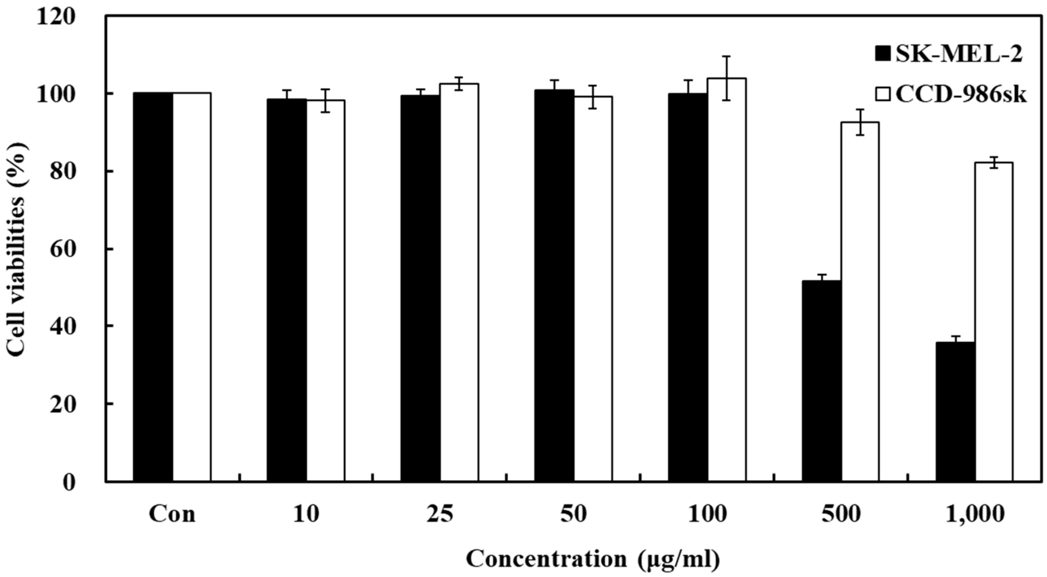


**Supplementary Figure S1.** Effects of PGG on cell viability in SK-Mel-2 and CCD-986sk cells. SK-Mel-2 human melanoma cells (1 x $10^5$ cells/well) and CCD-986sk human normal dermal fibroblast cells (5 x $10^3$ cells/well) were seeded in 96-well plates and incubated for 24 h. After incubation, the cells were treated with different concentrations of PGG. Cell viability was measured using the MTT assay. The data are presented as the mean ± SD of three independent experiments. Con: control, PGG: pentagalloylglucose.

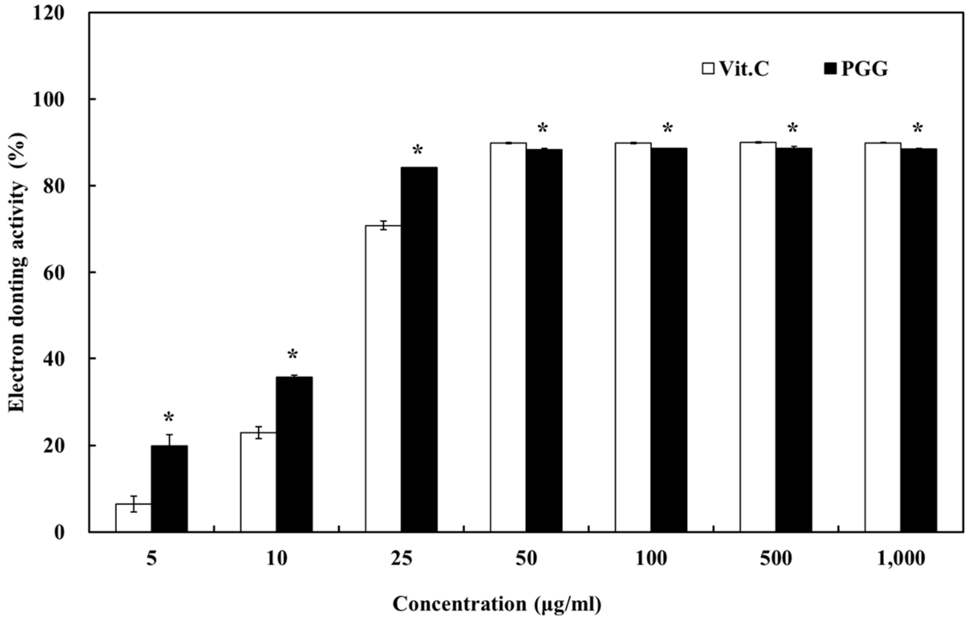


**Supplementary Figure S2.** Electron donating activity of extracts from PGG. The electron-donating ability was evaluated using the DPPH reagent. The samples and reagent were mixed in a 1:6 ratio and allowed to react for 15 min (in the dark). After the reaction, the absorbance was measured at 517 nm. All results are the mean ± standard deviation of triplicate determinations. (*$p$ < 0.05 vs Vit. C). PGG: pentagalloylglucose, Vit. C: L-ascorbic acid.

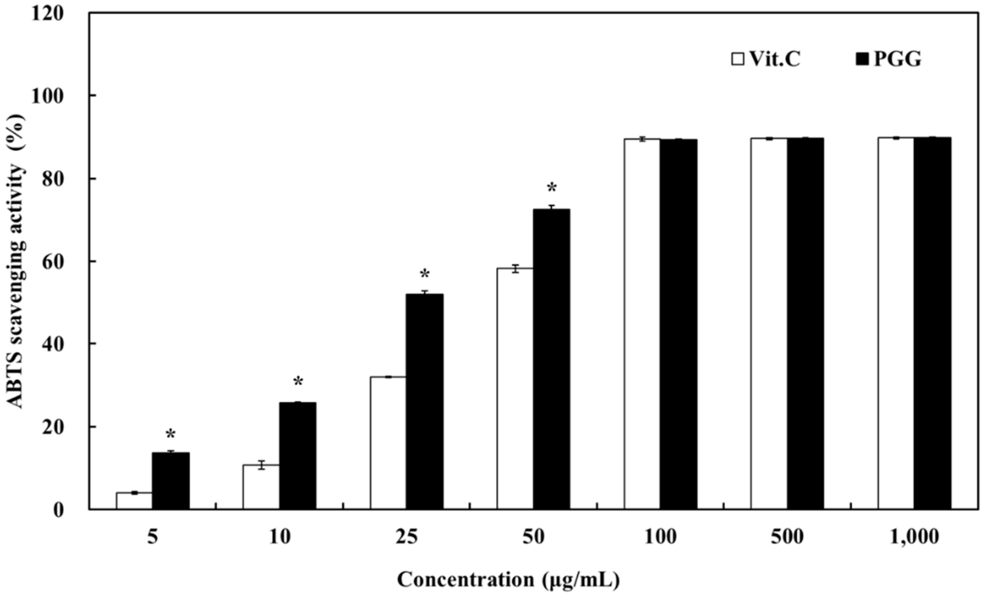


**Supplementary Figure S3.** ABTS radical scavenging ability of extracts from PGG. The ABTS radical scavenging activity was measured by mixing the ABTS solution and the sample in a 1:1 ratio. The decolorization of the solution indicated the scavenging of ABTS radicals. The absorbance was measured at 700 nm to quantify the radical scavenging efficiency. All results are the mean ± standard deviation of triplicate determinations. (*$p < 0.05$ vs Vit. C). PGG: pentagalloylglucose, Vit. C: L-ascorbic acid.
